# Contact tracing performance during the Ebola epidemic in Liberia, 2014-2015

**DOI:** 10.1371/journal.pntd.0006762

**Published:** 2018-09-12

**Authors:** Krista C. Swanson, Chiara Altare, Chea Sanford Wesseh, Tolbert Nyenswah, Tashrik Ahmed, Nir Eyal, Esther L. Hamblion, Justin Lessler, David H. Peters, Mathias Altmann

**Affiliations:** 1 Liberia Country Office, Action Contre la Faim, Paris, France; 2 Public Health Emergencies, Liberia Ministry of Health, Monrovia, Liberia; 3 Bloomberg School of Public Health, Johns Hopkins University, Baltimore, Maryland, United States of America; 4 Harvard T.H. Chan School of Public Health, Harvard University, Boston, Massachusetts, United States of America; 5 Liberia Country Office, World Health Organization, Monrovia, Liberia; Institute for Disease Modeling, UNITED STATES

## Abstract

**Background:**

During the Ebola virus disease (EVD) epidemic in Liberia, contact tracing was implemented to rapidly detect new cases and prevent further transmission. We describe the scope and characteristics of contact tracing in Liberia and assess its performance during the 2014–2015 EVD epidemic.

**Methodology/Principal findings:**

We performed a retrospective descriptive analysis of data collection forms for contact tracing conducted in six counties during June 2014–July 2015. EVD case counts from situation reports in the same counties were used to assess contact tracing coverage and sensitivity. Contacts who presented with symptoms and/or died, and monitoring was stopped, were classified as “potential cases”. Positive predictive value (PPV) was defined as the proportion of traced contacts who were identified as potential cases. Bivariate and multivariate logistic regression models were used to identify characteristics among potential cases.

We analyzed 25,830 contact tracing records for contacts who had monitoring initiated or were last exposed between June 4, 2014 and July 13, 2015. Contact tracing was initiated for 26.7% of total EVD cases and detected 3.6% of all new cases during this period. Eighty-eight percent of contacts completed monitoring, and 334 contacts were identified as potential cases (PPV = 1.4%). Potential cases were more likely to be detected early in the outbreak; hail from rural areas; report multiple exposures and symptoms; have household contact or direct bodily or fluid contact; and report nausea, fever, or weakness compared to contacts who completed monitoring.

**Conclusions/Significance:**

Contact tracing was a critical intervention in Liberia and represented one of the largest contact tracing efforts during an epidemic in history. While there were notable improvements in implementation over time, these data suggest there were limitations to its performance—particularly in urban districts and during peak transmission. Recommendations for improving performance include integrated surveillance, decentralized management of multidisciplinary teams, comprehensive protocols, and community-led strategies.

## Author summary

Contact tracing is comprised of three main steps: identifying, listing, and monitoring persons who have been exposed to infected individuals, with the goal of rapidly diagnosing and treating new cases and preventing further spread of infection. This approach has been used to control transmission of infectious diseases including smallpox, tuberculosis, HIV, and syphilis, and while contact tracing has been used in prior outbreaks of hemorrhagic fever, these outbreaks were small in scale. During the 2014–2015 Ebola virus disease (EVD) epidemic in Liberia, contact tracing was implemented in all 15 counties on a scale that was unprecedented, particularly within both rural and crowded urban settings. This work provides insight into the magnitude that which contact tracing was implemented, its characteristics, as well as an assessment on its performance. Given that contract tracing is a critical tool for controlling disease spread, these findings aid in informing future planning and decision making for its implementation.

## Introduction

In March 2014, Liberia detected its first cases of Ebola virus disease (EVD) in Lofa, a northern county bordering Guinea and Sierra Leone [1]. The Liberian Ministry of Health (MOH) (formerly Ministry of Health and Social Welfare) established a national task force and initiated control efforts, including contact tracing [1, 2]. As the epidemic grew, the task force developed into an Incident Management System, which oversaw contact tracing in all 15 counties with support from international partners including World Health Organization (WHO), U.S. Centers for Disease Control and Prevention (CDC), and Action Contre la Faim [3, 4, 5]. Continuous, widespread transmission continued until February 2015 [6], and 42 days after the last confirmed case had two negative samples in March 2015 [7], Liberia was declared free of Ebola on May 9, 2015—marking an end to the epidemic [8].

Contact tracing is comprised of three main steps: identifying, listing, and monitoring persons who have been exposed to infected individuals, with the goal of rapidly diagnosing and treating new cases and preventing further spread of infection. This approach has been used to control transmission of infectious diseases including smallpox, tuberculosis, HIV, and syphilis [9, 10, 11, 12]. Although contact tracing has been used in prior outbreaks of hemorrhagic fever, these outbreaks were small in scale [13, 14]. Contact tracing is most efficient for diseases with low incidence, limited transmissibility [15, 16], tight networks, and an incubation period long enough to allow intervention. Conversely, the effectiveness and optimal levels of investment for contact tracing, particularly for emerging diseases and for acute epidemics, are subjects of ongoing research and debate [15, 16, 17, 18, 19].

The 2014 EVD epidemic was the largest EVD outbreak in history and the first known EVD outbreak in West Africa [20]. The scale of the control efforts including contact tracing was unprecedented, particularly within both rural and crowded urban settings, which burdened existing surveillance capabilities and required immense commitment and cooperation on the part of government and the affected communities themselves. Furthermore, the strategies and implementation of contact tracing in Liberia evolved—from establishing operations to scaling them up—in order to respond to the changing phases of the epidemic. These aspects warrant the need to further examine contact tracing within this unique context. Here, we describe the scope and characteristics of contact tracing in Liberia and explore its performance during the 2014–2015 EVD epidemic in order to inform future contact tracing strategies in large-scale epidemics.

## Methods

### Study design and setting

We performed a retrospective descriptive analysis of data collection forms for contact tracing that was conducted for the EVD epidemic in six of Liberia’s 15 counties during June 2014–July 2015. The six counties consisted of both rural and urban areas and represented 72% of the population of Liberia [21]. Three of the counties (Lofa, Bong, and Nimba) are at the border with Cote d’Ivoire, Guinea, or Sierra Leone, while the other three (Montserrado, Margibi, and Sinoe) extend from central areas to the coast. Additionally, both formal and informal sources of information regarding contact tracing organizational structures and implementation within these counties were reviewed to help provide context for the data analysis.

### Contact definition

A contact was defined as a person who had direct or indirect exposure to any confirmed, probable, or suspect EVD case, or bodily fluids of a case, within the past 21 days [22, 23]. This definition also included any persons who had been discharged from an Ebola Treatment Unit (ETU) as not a case, due to their potential exposure to the virus while in the ETU.

### Contact tracing procedures

National contact tracing guidelines and forms, which were initially adapted from existing WHO and CDC materials and finalized during the waning days of the epidemic, were used as the foundation for implementing the three steps of contact tracing: contact identification, listing, and monitoring. Once a case was detected, contact identification and listing were conducted by interviewing the case and/or family members to gather an initial list of potentially exposed persons. In most instances, this process was conducted by case investigation teams, which were distinct from contact tracing teams, and any of the following six types of exposure were added: (1) sleeping or eating in the same household; (2) direct physical contact with the body; (3) touching bodily fluids; (4) manipulating clothes or other objects; (5) through breastfeeding; and (6) attending a case’s funeral.

Contact tracers, chosen from within the community, located the listed contacts and identified any additional contacts missed in the initial investigation. Contact tracers transferred the information collected by case investigation teams to paper forms, including the contact’s name, county, district, town, and exposure(s). The name, age, location, and unique case identifier of the case for which contacts were listed, i.e. the “source case”, were also recorded.

During contact monitoring, contact tracers were expected to visit contacts twice daily (morning and afternoon) for 21 days post-exposure in order to identify and record whether the contact had EVD symptoms. This was determined initially through self-reports and physical observation, and eventually temperature readings were added for more objective monitoring. Contacts were monitored for nine symptoms: joint pain, fever (>38° Celsius), weakness, nausea, diarrhea, headache, throat pain, red eyes, and mucosal bleeding.

### Data analysis

Following the outbreak, paper contact tracing forms were requested from all County Health Teams. Forms were received from six counties and the data were entered into a Microsoft Access database. Data were analyzed using Microsoft Access and Epi Info. Each form was considered a unique contact record and unit of analysis, though it was possible for individual contacts to be monitored more than once if re-exposed.

Source cases were identified using unique case identifiers, name, age, county, and district. To assess the coverage of contact tracing, or the percentage of cases for which contacts were monitored, we calculated the ratio of source cases in the database to the total number of suspected, probable, and confirmed EVD cases in MOH situation reports for the same counties using the closest approximate dates [24, 25, 26, 27, 28]. The mean number of contacts per source case was presented as contact-to-case ratios.

We analyzed records by county and district. Urban or rural classifications were assigned based on districts; districts that hold the county headquarters or that have settlements with a population of 5,000 or more persons were classified as urban [21]. Two districts, one each in Nimba and Montserrado counties, were divided into urban and rural sub-districts. Each of the six exposure categories and nine symptoms were analyzed, and medians and interquartile ranges (IQR) were calculated.

We divided the timeframe into four phases based on the observed epidemic trends of cases within Liberia [5], per epidemiologic week (EW): “Phase 1”: the initial increase of cases, from June to mid-August 2014 (EW 22–33); “Phase 2”: the peak, from mid-August to mid-November 2014 (EW 34–46); “Phase 3”: a decline in the epidemic, from mid-November 2014 through February 2015 (EW 47–9); and “Phase 4”: sporadic clusters, from March through July 2015 (EW 10–31). The first date of contact monitoring or last date of exposure was used to categorize records by phase. Medians and IQRs for timeliness, determined by the difference between the last date of exposure and first date of follow-up, were calculated and stratified by urban-rural and phases.

Each record was assigned one of seven outcomes of monitoring, either designated on the form or imputed using supplemental information: (1) “completed” the monitoring period of 21 days post-exposure; (2) “dropped” if the source was determined to be not a case; (3) “lost to follow-up” if the contact could not be located after three consecutive days; (4) “potential cases” if the contact presented with symptoms and/or died and monitoring was stopped; (5) “restarted” if monitoring was reinitiated due to a new exposure; (6) “transferred” if the contact moved to another jurisdiction; or (7) “unknown” for all remaining contacts with no outcome information. Contacts who presented with symptoms could be referred for medical evaluation without meeting EVD case definitions; hence, we use the terminology “potential cases”.

We calculated the positive predictive value (PPV) defined as the proportion of traced contacts—excluding those with dropped and unknown outcomes—who were potential cases. Sensitivity was defined as the ratio of potential cases identified during monitoring to the number of new cases in situation reports in the same counties [24, 25, 26, 27, 28]. This analysis assumes all potential cases were infected with EVD, and that all source cases and potential cases in the database were included in the total counts from situation reports. Therefore, to the extent that these assumptions are overstated, the calculations serve as upper limit estimates. PPVs were stratified by urban-rural and epidemic phases, whereas sensitivity and coverage were stratified by phases.

We used odds ratios and 95% confidence intervals to examine exposure types, symptom types, phases, and urban-rural amongst potential cases compared with contacts who completed monitoring; for ordinal variables, the lowest category was used as a reference group. Chi-square tests with p-values <0.05 were statistically significant. Two multivariate logistic regression models were used: (1) urban-rural, phase, and exposure type covariates, limited to records with ≥1 exposures, and (2) urban-rural, phase, and symptom type covariates, limited to records with ≥1 symptoms. Only statistically significant variables in bivariate analysis were included in the models. Nonparametric tests were used for continuous variables.

### Ethical considerations

This assessment is included under Johns Hopkins School of Public Health Institutional Review Board no. 6296 with DHP as principal investigator. A letter of agreement was signed with the Liberia MOH concerning the publication of contact tracing analyses. This assessment used retrospective data collected for public health surveillance purposes so informed consent was deemed unnecessary according to the U.S. Common Rule. We followed the Declaration of Helsinki, aiming to provide assurance that the rights, integrity, and confidentiality of participants were protected.

## Results

### Source cases

We analyzed 25,830 records for contacts who had monitoring initiated or were last exposed between June 4, 2014 and July 13, 2015 in the six counties. Of these, 25,651 contacts were listed for 2,465 source cases; an additional 179 contacts had no source case provided. The overall contact-to-case ratio was 10:1 (median = 7, range 1–424). The contact-to-case ratio increased with each subsequent phase and was higher in urban than rural districts. There were 9,241 EVD cases in situation reports in the six counties. The upper limit estimate of coverage, or the maximum percentage of cases for which contacts were monitored, was 26.7%, and was lowest during Phase 1. (Table 1)

**Table 1:** Performance indicators overall and by urban-rural and phases, six counties—Liberia, June 2014–July 2015.

	Overall	Urban	Rural	Phase 1	Phase 2	Phase 3	Phase 4
Listed contacts with a source case	N = 25,651	N = 21,369	N = 4,282	N = 821	N = 15,784	N = 8,348	N = 561
Source cases	2,465	1,874	647	144	1,703	587	30
Contact-case ratio	10:1	11:1	7:1	6:1	9:1	14:1	19:1
Total EVD cases	N = 9,241	N/A	N/A	N = 796	N = 6,397	N = 8,291	N = 9,241
% Coverage	26.7	N/A	N/A	18.1	28.9	29.4	26.7
Timeliness	N = 25,300	N = 21,178	N = 4,119	N = 791	N = 15,402	N = 8,405	N = 562
Median days (IQR)	1 (0–4)	1 (1–4)	0 (0–2)	1 (0–7)	1 (0–3)	1 (1–4)	1 (1–3)
Lost contacts*	N = 25,569	N = 21,271	N = 4,295	N = 794	N = 15,701	N = 8,387	N = 562
No. (%)	136 (0.5)	112 (0.5)	24 (0.6)	9 (1.1)	102 (0.7)	25 (0.3)	0 (0.0)
PPV^†^	N = 23,801	N = 19,679	N = 4,119	N = 767	N = 15,638	N = 7,012	N = 261
No. (%)	334 (1.4)	212 (1.1)	119 (3.0)	36 (4.7)	252 (1.6)	37 (0.5)	0 (0.0)
New EVD cases	N = 9,241	N/A	N/A	N = 796	N = 5,601	N = 1,892	N = 943
% Sensitivity	3.6	N/A	N/A	4.5	4.5	2.0	0.0

*Excludes contacts with an unknown outcome.

^†^Excludes contacts that were dropped or with an unknown outcome.

### Spatiotemporality

**Fig 1:**
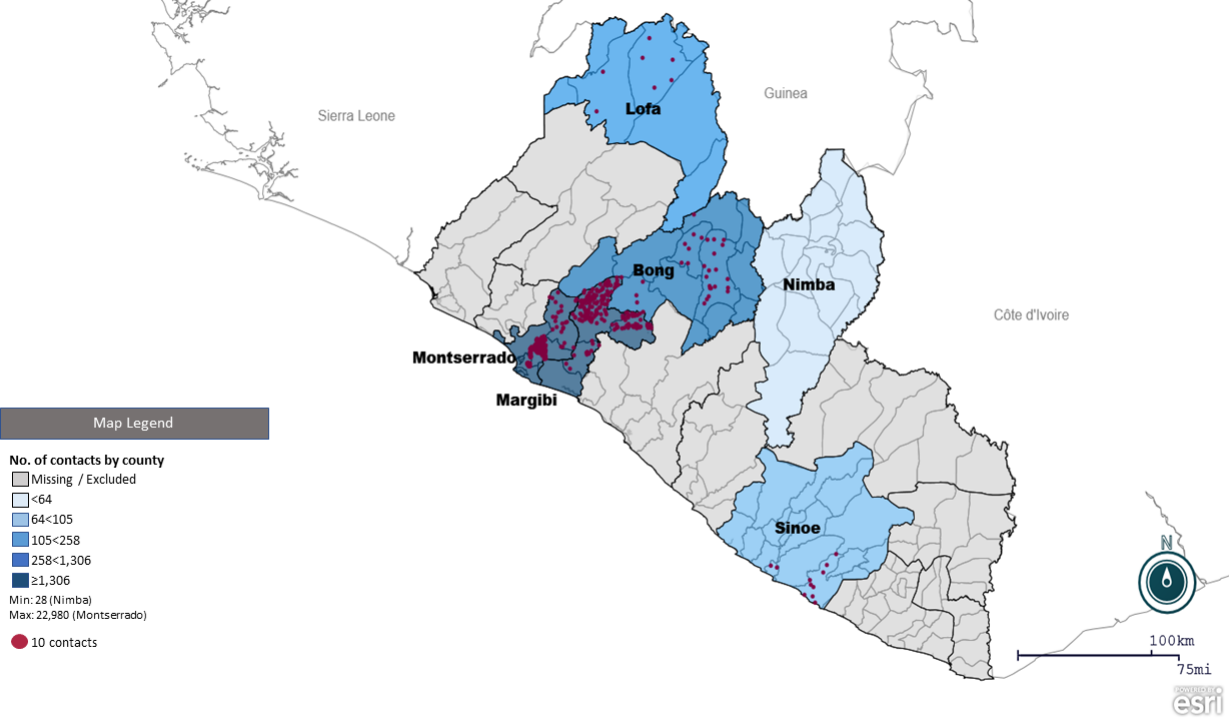
Geographical distribution of monitored contacts by county (choropleth) and by district (dot density*)—Liberia, June 2014–July 2015. Map created using Epi Info. *1 dot = 10 contacts.

In the six counties providing data, 89.0% of the records were identified in Montserrado County, 8.6% in Margibi, 1.6% in Bong, 0.4% in Lofa, 0.4% in Sinoe, and 0.1% in Nimba (Fig 1) (Table 2). Records pertained to 22 of Liberia’s 136 districts (Table 2); data from the remaining districts was unavailable due to no contact tracing records or no reported EVD cases. In total, 21,500 (83.2%) contacts were in seven urban districts/sub-districts, mainly in the Monrovia capital district in Montserrado, while 4,327 (16.8%) contacts were in 17 rural districts/sub-districts. Potential cases were less likely to be from urban districts (Table 3).

**Table 2:** Contact tracing records by county—Liberia, June 2014–July 2015.

County	County population	Contact tracing records	Listed districts
		n/N (%)	n/N (%)
Montserrado	1,118,241	22,980/25,830 (89.0)	5/5 (100.0)
Margibi	209,923	2,208/25,830 (8.6)	4/4 (100.0)
Bong	333,481	404/25,830 (1.6)	7/12 (58.3)
Lofa	276,863	111/25,830 (0.4)	3/7 (42.9)
Sinoe	102,391	99/25,830 (0.4)	2/17 (11.8)
Nimba	462,026	28/25,830 (0.1)	1/17 (5.9)

**Table 3:** Characteristics among contacts completing monitoring versus potential cases, six counties—Liberia, June 2014–July 2015.

	All outcomes	Completed	Potential cases	Potential cases vs. Completed	Potential cases vs. Completed
	n/N (%)	n/N (%)	n/N (%)	OR (95% CI)	aOR (95% CI)^‡^
Urban-Rural					
Urban	21,500/25,827 (83.2)	18,777/22,680 (82.8)	212/331 (64.1)	0.37 (0.30–0.47)^†^	0.52 (0.38–0.70)^†^
Epidemic phase					
Phase 1	823/25,690 (3.2)	706/22,568 (3.1)	36/325 (11.1)	Ref	Ref
Phase 2	15,859/25,690 (61.7)	14,861/15,567 (95.5)	252/288 (87.5)	0.33 (0.23–0.48)^†^	0.19 (0.12–0.28)^†^
Phase 3	8,446/25,690 (32.9)	6,740/7,446 (90.5)	37/73 (50.7)	0.11 (0.07–0.17)^†^	0.07 (0.04–0.12)^†^
Phase 4	562/25,690 (2.2)	261/967 (27.0)	0/36 (0.0)	0.00 (0.00–∞)	
Exposure types per contact*					
Slept or ate in same household	8,654/17,876 (48.4)	7,827/16,277 (48.1)	146/211 (69.2)	2.42 (1.81–3.25)^†^	2.16 (1.59–2.94)^†^
Direct physical contact with body	13,064/17,876 (73.1)	11,884/16,277 (73.0)	173/211 (82.0)	1.68 (1.18–2.40)^†^	1.76 (1.21–2.54)^†^
Touched bodily fluids	5,454/17,876 (30.5)	4,975/16,277 (30.6)	95/211 (45.0)	1.86 (1.42–2.45)^†^	1.52 (1.13–2.05)^†^
Manipulated clothes or objects	6,701/17,876 (37.5)	6,052/16,277 (37.2)	103/211 (48.8)	1.61 (1.23–2.12)^†^	1.33 (0.99–1.80)
Breastfed a child	30/17,876 (0.2)	28/16,277 (0.2)	0/211 (0.0)	0.00 (0.00–∞)	
Attended funeral	381/17,876 (2.1)	379/16,277 (2.3)	1/211 (0.5)	0.00 (0.00–∞)	
No. exposure types per contact*					
Single exposure	8,065/17,876 (45.1)	7,387/16,277 (45.4)	52/211 (24.6)	Ref	
Two exposures	5,340/17,876 (29.9)	4,850/12,237 (39.6)	56/108 (51.9)	1.64 (1.12–2.40)^†^	
Three exposures	2,365/17,876 (13.2)	2,119/9,506 (22.3)	58/110 (52.7)	3.89 (2.67–5.67)^†^	
Four exposures	2,088/17,876 (11.7)	1,906/9,293 (20.5)	45/97 (46.4)	3.35 (2.24–5.02)^†^	
Five exposures	16/17,876 (0.1)	13/7,400 (0.2)	0/52 (0.0)	0.00 (0.00–∞)	
Six exposures	2/17,876 (0.0)	2/7,389 (0.0)	0/52 (0.0)	0.00 (0.00–∞)	

*Denominator is limited to contacts reporting ≥1 exposure types.

^†^Statistical significance (p<0.05).

^‡^Multivariate analysis adjusting for urban-rural, phase, and exposure type covariates; only variables that were statistically significant in bivariate analysis, and denominator is limited to contacts reporting ≥1 exposure types (N = 16,470).

**Fig 2:**
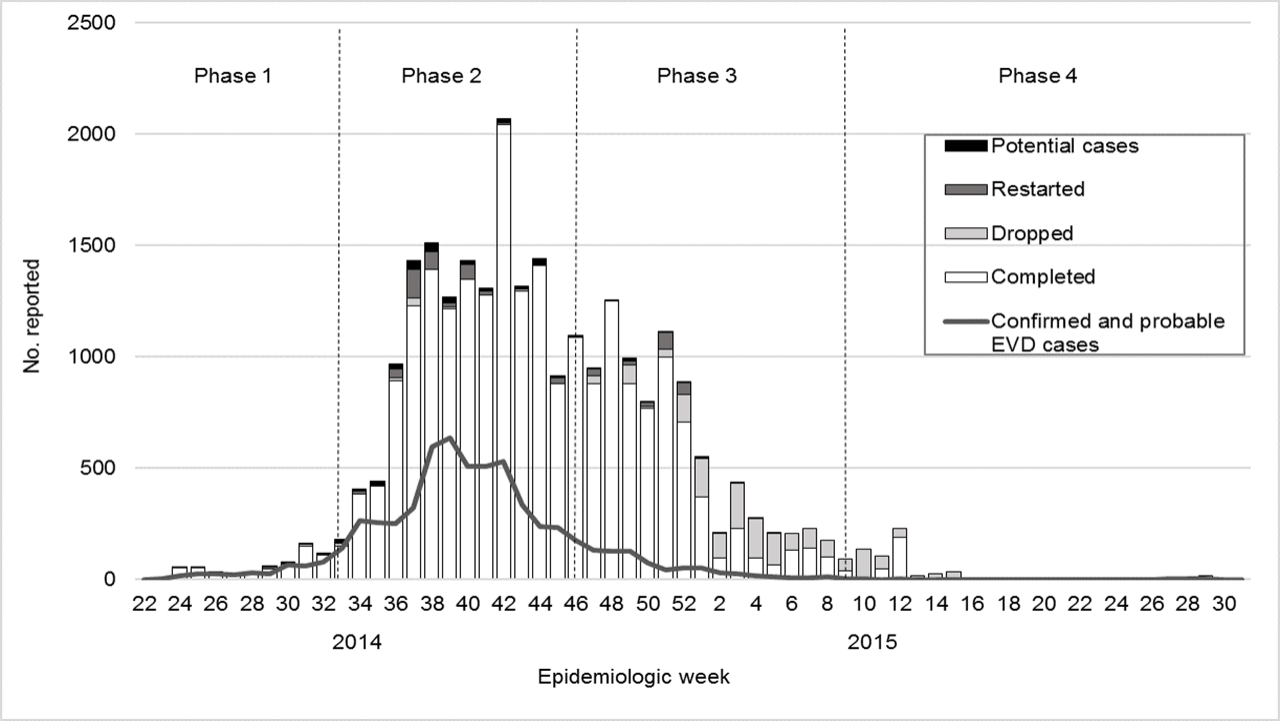
Contacts in six counties* and EVD cases^†^, by epidemiologic week—Liberia, June 2014–July 2015 *By outcome group (N = 25,296); excludes contacts that were transferred, lost to follow-up, or with an unknown outcome. ^†^National confirmed and probable EVD cases reported in situation reports (N = 6,035) [34].

Temporal trends for contact tracing aligned with disease transmission trends (Fig 2). For 25,690 records grouped by phase, 61.7% were monitored during Phase 2 and 32.9% during Phase 3. Only contacts in Montserrado were monitored during Phase 4. No contacts were monitored during May–June 2015, corresponding to the Ebola-free period. Based on 25,300 records, contact tracing was timelier in rural districts; overall, the median difference was 1 day (IQR 0–4) (Table 1).

### Exposures

Of 25,830 total contacts, 17,876 (69.2%) contacts reported 34,284 exposure types, and 7,954 (30.8%) had zero exposures recorded. Among the 17,876 contacts reporting any exposure, direct physical contact with the body was the most common (73.1%), while funeral attendance (2.1%) and breastfeeding (0.2%) were the least common (Table 3). Two or more exposure types were reported in 54.9% of 17,876 contacts; the median was 2 (IQR 1–3). Multivariate analysis showed the odds of sleeping or eating in the same household, direct physical contact, or touching bodily fluids were higher amongst potential cases than contacts completing monitoring (Table 3).

### Outcome

Of 25,569 contacts with an assigned outcome, 22,680 (87.8%) completed monitoring, 1,768 (6.8%) were dropped, 637 (2.5%) restarted, 334 (1.3%) were potential cases, 136 (0.5%) were lost to follow-up, and 14 (0.1%) were transferred. Most contacts completed monitoring during each phase except during Phase 4, when 53.6% of contacts were dropped (Fig 2). More contacts restarted during phases 2 and 3 than other phases. Potential cases were less likely to be monitored during phases 2 or 3 compared to Phase 1 (Table 3). Twenty-two contacts were not located prior to monitoring. Of 46 recorded contact deaths, 56.5% were in urban districts and 33 occurred during monitoring (15 after taken to an ETU).

The PPV was 1.4% overall, and was higher in rural (3.0%) than urban (1.1%) districts and highest during Phase 1 (4.7%), after which it decreased for subsequent phases. The sensitivity of monitoring, or the maximum proportion of new cases detected, was 3.6%, and was highest during phases 1 and 2. (Table 1)

### Symptoms

Table 4 shows the distribution of reported symptoms. Overall, 326 contacts reported 1,299 symptom types and 3,732 symptom-days; the median symptom types per contact was 4 (IQR 2–5). Contacts of all outcomes reported symptoms except transferred contacts; 218 (66.9%) of 326 contacts reporting symptoms were potential cases, 92 (28.2%) completed monitoring, 6 (1.8%) restarted, 6 (1.8%) were unknown, 3 (0.9%) were dropped, and 1 (0.3%) was lost to follow-up. In multivariate analysis, potential cases were more likely to report fever, nausea, or weakness compared with contacts who completed monitoring.

**Table 4:** Symptoms among contacts completing monitoring versus potential cases, six counties—Liberia, June 2014–July 2015.

	All outcomes	Completed	Potential cases	Potential cases vs. Completed	Potential cases vs. Completed
	n/N (%)	n/N (%)	n/N (%)	OR (95% CI)	aOR (95% CI)
Symptom types per contact*					
Joint pain	185/326 (56.8)	37/92 (40.2)	140/218 (64.2)	2.67 (1.62–4.40)^†^	1.26 (0.67–2.36)
Fever	254/326 (77.9)	56/92 (60.9)	187/218 (85.8)	3.88 (2.20–6.83)^†^	2.24 (1.12–4.49)^†^
Weakness	237/326 (72.7)	45/92 (48.9)	182/218 (83.5)	5.28 (3.07–9.09)^†^	2.65 (1.38–5.11)^†^
Nausea	139/326 (42.6)	14/92 (15.2)	119/218 (54.6)	6.70 (3.57–12.55)^†^	4.34 (1.99–9.48)^†^
Diarrhea	112/326 (34.4)	15/92 (16.3)	93/218 (42.7)	3.82 (2.07–7.06)^†^	1.05 (0.47–2.32)
Headache	214/326 (65.6)	57/92 (62.0)	147/218 (67.4)	1.27 (0.77–2.11)	
Throat pain	68/326 (20.9)	14/92 (15.2)	50/218 (22.9)	1.66 (0.87–3.18)	
Red eyes	68/326 (20.9)	12/92 (13.0)	53/218 (24.3)	2.14 (1.08–4.23)^†^	0.94 (0.41–2.11)
Bleeding	22/326 (6.8)	7/92 (7.6)	15/218 (6.9)	0.90 (0.35–2.28)	
No. symptom types per contact*					
Single symptom	57/326 (17.5)	36/92 (39.1)	16/218 (7.3)	Ref	
Two symptoms	41/326 (12.6)	15/51 (29.4)	24/40 (60.0)	3.60 (1.50–8.62)^†^	
Three symptoms	46/326 (14.1)	12/48 (25.0)	32/48 (66.7)	6.00 (2.47–14.57)^†^	
Four to nine symptoms	182/326 (55.8)	29/65 (44.6)	146/162 (90.1)	11.33 (5.56–23.06)^†^	

*Denominator is limited to contacts reporting ≥1 symptom types.

^†^Statistical significance (p<0.05).

^‡^Multivariate analysis adjusting for urban-rural, phase, and symptom type covariates; only variables that were statistically significant in bivariate analysis were included, and denominator is limited to contacts reporting ≥1 symptom types (N = 308).

## Discussion

During 2014–2015, more than 25,000 persons in six of Liberia’s 15 counties were identified, listed, and monitored for EVD, representing one of the largest contact tracing efforts during an epidemic in history. Nationwide, these efforts were even more substantial and required the dedication of responders, including the Government of Liberia, counties, and contact tracing teams. As a result, 334 contacts were identified as potential cases with the intention of providing earlier treatment and preventing hundreds of new infections.

Relative to the scale of these efforts, however, these data suggest there were limitations to the performance of contact tracing within Liberia. Overall, there was a small proportion of monitored contacts that were identified as potential cases, and more than 97% of reported EVD cases from the six counties were not detected through contact monitoring. This is greater than expected, especially compared to other examples in West Africa where approximately 69% to 78% of cases were not being traced prior to case identification [29, 30]. This measure is dependent upon the level of contact tracing coverage, and based on our database, though admittedly not comprehensive, coverage only accounted for a maximum of one-quarter of all EVD cases reported for these six counties. While this ratio is aligned with similar findings in two Guinea prefectures (32% and 39%) and in Sierra Leone (19%) [29, 30], it is possible that contact tracing was not initiated for up to three-quarters of the remaining EVD cases in Liberia, potentially due to a combination of factors discussed below.

Potential cases were more likely to be identified in rural districts and early in the epidemic, despite intensified efforts as the epidemic progressed. Possible explanations for why contact tracing was less effective in urban areas could include the following: higher population density and complex social networks making it more difficult to identify all contacts; less cooperation within urban settings; higher burden and strained resources; or a combination of these factors. These results support the concept that contact tracing is most successful when transmission is low, and models have shown that expanding implementation of contact tracing yields diminishing reductions in disease prevalence [15, 16]. Therefore, it is critical to conduct contact tracing rigorously and comprehensively as soon as an outbreak is identified, and to achieve higher sensitivity and coverage during this phase.

There were, however, notable improvements in implementation over time; specifically, greater coverage, fewer contacts lost to follow-up, and higher contact-to-case ratios. During Phase 4, Liberia was able to focus more resources on eliminating the last transmission chains, including expanding the inclusion criteria to ensure no new cases went undetected [6]. This would have resulted in a larger contact-to-case ratio during Phase 4 compared to all other phases.

The dynamics of contact tracing are complex, and its success is related to characteristics of the disease and etiologic agent, resources, and socio-political factors that influence its acceptability and implementation. Additionally, the approaches to contact tracing may differ depending on whether there is a vaccine or therapy available. Given that contact tracing remains one of the critical public health tools during outbreaks involving person-person transmission, optimizing its performance is paramount. While not exhaustive, we focus on four key challenges that may have limited the performance of contact tracing for EVD within Liberia, and propose recommendations for future efforts.

First, an integrated surveillance and data management system was lacking and had to be established for reporting between the national laboratory, healthcare facilities and ETUs, and contact tracing and case investigation field teams [5]. Consequently, contact tracing was less functional at the beginning of the epidemic when it could have been most effective in slowing the epidemic. Initially, an insufficiently integrated system resulted in missed source cases and contacts, and led to delays in monitoring; this is reflected in that 25% of contacts with available information started monitoring four days after their last exposure. Additionally, contacts were listed and needlessly traced because of delays in receiving negative laboratory results, thereby lowering the PPV. Although mobile applications had the potential to improve reporting and data management, these were not piloted until after the peak of the outbreak. In contrast, contact tracing in urban Nigeria successfully and rapidly contained EVD transmission, largely thanks to robust surveillance systems and leveraging mobile applications for real-time monitoring [31, 32]. Strengthening integrated surveillance and electronic data systems, and the early adoption of mobile technology, could help improve timely reporting for listing and monitoring contacts.

Secondly, the organizational structure for contact tracing likely led to inefficiencies in its implementation and management, particularly in urban districts. For instance, case investigation teams, who conducted contact listing, were often distinct from contact tracing teams who conducted contact monitoring. In some rural areas, teams responded in tandem thereby reducing gaps, yet this was more difficult in dense urban areas such as in Montserrado County. Additionally, the county level coordinated all aspects of the response—not just contact tracing. In January 2015, Montserrado created decentralized sub-county sectors to oversee and synchronize all operations—a change previously recognized as a critical step for halting transmission [6]. Particularly in urban areas and in the absence of a robust surveillance system, using a decentralized management approach and multidisciplinary teams may improve contact tracing performance.

Thirdly, there were challenges with adapting and implementing contact tracing protocols, which had to be used by novice teams during the epidemic. For instance, the number of contacts per source case ranged widely in our analysis, from 1 to 424, and nearly one-third of contacts had no exposure documented, indicating that some contacts may not have met the inclusion criteria, thereby straining resources. Also, written guidance for identifying potential cases during monitoring did not specify how contact tracers should determine when to refer a contact for medical evaluation [23]. Eighteen contacts, who presumably would have shown symptoms prior to death, died during monitoring without being referred for medical evaluation. Among contacts who reported symptoms, including multiple symptoms and symptom-days, 33.1% continued under monitoring without being referred for further evaluation, indicating that triggers for identifying potential cases was subjective. During future outbreaks, clear and comprehensive protocols need to be initiated early in the epidemic and reinforced throughout implementation. Furthermore, if resources are limited, inclusion criteria could prioritize contacts with multiple exposures, and/or those with household contact or direct contact with the body or bodily fluids. Triggers for identifying potential cases could include contacts reporting multiple symptoms types, fever, nausea, and weakness.

Finally, community perceptions, stigma, and mistrust reportedly led to challenges in obtaining complete and reliable information, to delays or an inability to trace contacts due to evasion, and even to violence [5, 33]. Underreporting of symptoms due to fear or due to fever-reducing drugs may explain why relatively few symptoms were captured in our database. Also, contacts were instructed to self-isolate within their home, which disrupted normal routines and the ability to maintain jobs; without adequate support from the community or organizations, contacts are less likely to cooperate. These aspects stress the importance of community cooperation, trust, and engagement. Overall, less than 1% of contacts were lost to follow-up, and this improved during each phase along with more contacts listed per source case, suggesting that this cooperation probably improved as the outbreak progressed. For future outbreaks, community-led strategies for contact tracing should be an early priority to foster cooperation, trust, and ownership of the control efforts.

This analysis represented both urban and rural settings, and Montserrado specifically, where the response was most intense. However, we were unable to collect forms from all 15 counties nor all forms from the six inclusive counties; for example, no forms were available for the EVD cluster that occurred in Margibi in July 2015. Despite commendable efforts, counties reported that paper forms were lost or destroyed due to perceived contamination risks. Using paper forms also led to variability in data quality, including illegible writing, misspellings, inversed source case and contact information, and difficulty in interpreting marks for visits and the presence of symptoms. Falsifying information on forms was a concern [33], such as documenting visits when the contact had not been seen, and this was an issue early in the epidemic.

These factors, combined with the lack of information to ascertain the final status of EVD infection amongst source cases and potential cases, constrained our analysis. Likewise, we could not conclude whether symptoms reported amongst potential cases evidenced EVD infection. Our data primarily represented contact monitoring, as we did not have a comprehensive contact listing. Finally, EVD case counts from situation reports were unavailable to stratify coverage and sensitivity by urban-rural districts and/or phase, and these aggregated totals could not be linked to our individual-level database.

Our findings suggest that despite the unprecedented scale of contact tracing for EVD in Liberia, there were limitations in its ability to detect new cases, especially in urban areas and during the peak case load. Since contact tracing remains a critical intervention for controlling outbreaks, we suggest rigorous implementation early in the outbreak and focusing on four key areas to optimize its performance within similar contexts: (1) strengthening integrated surveillance and electronic data systems, (2) decentralizing management of multidisciplinary teams for improved coordination and oversight, (3) instituting and reinforcing clear and comprehensive protocols, and (4) adapting community-led strategies to foster cooperation, trust, and ownership.

## Supporting information

S1 Dataset Contact tracing analysis dataset—Liberia, 2014–2015. De-identified listing of contact tracing records used for analysis (N = 25,830).
(XLSX)
